# Lithotomy and Wounded Rectum

**Published:** 1835

**Authors:** Horatio G. Jameson

**Affiliations:** Professor of Surgery in the Cincinnati College


### Art. III.—Lithotomy and Wounded Rectum.

*Some Account of a case of Stone, attended with several remarkable circumstances—and in which the operation of Lithotomy was performed three times.* By Horatio G. Jameson, M. D., Professor of Surgery in the Cincinnati College.

My memory has been awakened to the circumstances of this very interesting case, by reading a notice of Mr. Copland's successful treatment of rupture, between the vagina and rectum, which notice will be seen in the present number of the Western Journal of the Medical and Physical Sciences.

The subject of this truly interesting case was the late worthy Judge Sprigg, of the United States Court. This gentleman was sorely afflicted for several years with symptoms of stone in the bladder. From the commencement of severe symptoms, he endeavored to obtain the best advice in his case, he, therefore, procured the attentions of several surgeons, some of whom I do not now recollect, but Dr. Physick, and Dr. Davidge, of Baltimore, were among the number. These and others sounded the patient, so as to be satisfied, at least, that they could not find a calculus. He underwent various plans of treatment, the particulars of none of which do I recollect, till he came into my hands.

It is not my intention to go into the minutiæ of the case, I shall therefore only notice some of the more striking or peculiar circumstances and symptoms. I sounded this gentleman repeatedly in various positions of the body, aiding my efforts by occasional distention of the bladder by warm water injections. For a long time (several years) the patient was wholly unable to void his urine, and was compelled to draw it off frequently, which he did by means of a large flexible metallic catheter—there were frequent and very painful contractions of the bladder. The patient had not, during the use of the catheter, been sensible at any time of touching a stone. During my soundings, I told the Judge I had perception of something like a stone twice, but so faint as by no means to enable me to say there was a stone; indeed, this inquiry was rendered very difficult, on account of a spasmodic working of the neck, or some other part of the bladder, by rubbing so as to produce a ringing scraping kind of sound, against the sound or catheter. There did not appear to be any symptoms indicating serious disease of the prostate, although both this gland, and the entire neck of the bladder, were considerably enlarged. Upon the whole the patient was suffering greatly, from all the usual symptoms of stone, and yet no stone could be detected.

I recommended the frequent injection of warm water into the bladder, on the view that if there might be stone, it must either be partially encysted, or pocketed in some part of the bladder; or in the absence of stone, the bladder might have acquired, from long irritation, a contracted and fixed form of the muscles, by which contraction and dilatation of that viscus is effected. In the first case, the gradual distention of the coats might tend to loosen a calculus so situated; and, in the second, afford relief by distention of the coats, and thus remove the morbid sensibility which caused the contraction and pain of the bladder, as we sometimes see in painful contractions of the sphincter ani, in which, from abrasion, or great irritability of the termination of the rectum, the muscle contracts, so as to cause the most exquisite pain, and in which cases separation or dilatation is the most certain and immediate means of obtaining relief.

After pursuing this course a few weeks, it fell in the Judge's way to call on the late Dr. Gill, of Hagerstown, who had acquired the reputation of a dexterous and successful operator for stone. It so happened that this gentleman discovered the stone plainly, so easily as to excite surprise, that he should be so much more successful in finding what could not be found by so many well practised hands; but to my mind there is not a shadow of doubt, that this greater good fortune was entirely owing to the altered condition of the bladder, from the employment of the warm water injections, by which the bladder had no doubt been gradually distended. Drs. Davidge and Wright could readily distinguish the stone after Judge S. returned to Baltimore to undergo the first operation.

This happy discovery induced Judge S. to employ Dr. G. to perform the operation for the stone, which none but himself could, prior to this period, discover. The patient, however, preferred coming to Baltimore, where he might have the advice of Dr. Davidge. The operation was performed in the presence of Drs. Davidge and Wright. Some calculi were extracted, number not recollected, but the operator had the misfortune to cut the rectum low down; another proof, we may suppose, that "the race is not always to the swift, nor the battle to the strong," since Dr. G., at that time, had acquired a high reputation as an operator.

The patient finding himself in a most deplorable condition, went on, as soon as circumstances would admit of it, to Dr. Physick, in a few weeks, I believe, after the operation.

After some considerable time, (some weeks,) this gentleman returned to Baltimore, and put himself under my care, now much inclined to, if not decided in the opinion, that among his misfortunes was that of having been, by a strange course of events, thrown out of my hands. Of what took place in Philadelphia I recollect but little, having made no notes of it, although the Judge made me fully acquainted, at the time, with all the circumstances. It may suffice to say, that I was informed that Dr. Physick repeated the operation, and took away a wineglass full of small calculi. The relief from the operation lasted but a few days, when he was shocked to find that he had gained nothing by the second operation. It had been his misfortune, to use his own language, "while he was intent upon obtaining the best skill of the country, to find that he had been, by some strange fatality, kept from obtaining the advantages expected from the most common operating surgeons."

Candor knows no distinction, and I shall not therefore abstain from expressing without enmity, or affection, as my opinion, that, if a proper operation had been performed, the patient would have been cured by such operation, followed with skillful attentions. The operation was performed in the usual way, excepting that the fistula in perineo, which existed after the first operation, was made a part of the lateral incision. Now I am perfectly persuaded, that if the incision had been begun at the opening in the perineum; and carried down the sphincter ani, so as to throw the whole of the fistulous opening into one wound, and thus relieve that wound from the evils arising from the contractions of the sphincter muscle, it would have healed up kindly, and thus removed one of the most deplorable cases within the pale of human suffering. That this is not an opinion formed at this late period, will be proved by the method of operation adopted by me afterwards in this case, several years ago. But in addition to this, it is clear to my mind, that the calculi were not all removed in either the second or first operation, and this was the confirmed opinion of my talented and observing patient.

Judge Sprigg determined on putting himself under my care before he left Philadelphia, but dispirited as he was in mind, and broken down in body, he could not consent to a third operation, which it was evident would alone afford a ray of hope of final recovery. To this his mind was made up, but he wished to regain a portion of strength, which would enable him to undergo another severe operation. Under this state of mind he resolved upon visiting the Berkley Springs, with a hope of improving his general health; but, as I had often predicted, the traveling, together with the deplorable condition to which he was now reduced, continued more and more to prostrate him. Seeing that death was advancing daily more near, he, with great difficulty returned to Hagerstown, after being some weeks at the Springs. Arrangements were made for me to meet him, for the purpose of giving him the chances of another operation at that place.

Previously to stating the Judge's condition at this time, it seems proper that I state what was his condition when he returned from Philadelphia. He could now discover evidence of stone at almost every introduction of the catheter, by which alone could he be relieved of the urine. At very short intervals he suffered from the most agonizing spasms of the bladder. His bowels were torpid, and required frequent aperients; whenever he had a passage the more liquid fæces would pass, with spasmodic violence, into the urethra, through the opening made into the rectum in the first operation; and, such was the arrangement of the parts that, notwithstanding a free opening in the perineum, the irritation given to the ejaculator muscles, by the stimulous of the fæces, was such that they were driven from the meatus urinarius with great violence—what with the extreme pain and disgust arising from this deplorable state of things, this gentleman's situation was truly wretched, for, in addition to what we have just detailed, the fæces would at the same time pass through the natural outlet, and through the opening in the perineum and end of the penis.

The patient having made his arrangements for my meeting him at Hagerstown, he with great difficulty reached this point, after enduring much suffering by the way. I now found him greatly emaciated, and suffering every half hour with agonizing spasms of the bladder, attended by incessant hiccup, also peculiarly spasmodic, and so loud as to be heard out of doors, as the patient lay up stairs; there was now extreme debility, and inability to take almost any food. Such, indeed, was the state of the case, that I unhesitatingly told him that I could not allow myself to believe that the operation could now afford a prospect of relief, it being even doubtful whether his strength would enable him to undergo the operation, without risk of dying in our hands.

After careful reflection I was called to the bed side, when my patient expressed himself to this effect: I am a most afflicted sufferer; I strongly desire that an operation may be performed, under the hope of obtaining even temporary relief of short duration; you, in whom I have much confidence, and after you have encountered the fatigue of a long journey, desire me not to undergo the operation, to insist upon it, under such circumstances, would be little better than suicide, what then shall I do? ever so short a respite would be acceptable, nor would the operation be attended with more severe pain than I am almost constantly suffering.

Placed in these trying circumstances, and aware that Dr. Dorsey and Dr. Reynolds, who were present, preferred leaving the decision entirely to myself, I requested further advice, provided there was any within reach which was thought worth obtaining. Dr. Harrison, of Martinsburg, Va., had spent some time at the Springs, with Judge Sprigg, and had paid some attention to his case; had obtained the Judge's confidence, no doubt deservedly. Dr. H. was immediately sent for.

The consultation having been assembled, Dr. Harrison at once gave as his opinion, that we ought to comply with the Judge's wishes, inasmuch as he could not possibly loose by the operation, and from it there was a possibility of relief; without its speedy performance a lingering, painful death must terminate the case. Sustained then by the opinion of Dr. H., and supported by Dr. Reynolds, and Dr. Dorsey, I at once proceeded to the operation.

I had a capacious urethra, a fistula in perineo, and a fistulous opening between the urethra and rectum, just behind the sphincter ani, to deal with. However little chance of recovery, this operation should be so directed as to obtain, if possible, a cure of these fistulæ—if this can be effected without prolonging or increasing the sufferings of my patient, duty requires that my measures be so directed.

It fortunately occurred to my mind, that by proceeding so as to include these fistulæ in my incisions, I should effect all that was requisite to cure these annoying sores. A straight director was introduced into the anus, through the recto-urethral fistula; and its end brought out through the opening in the perineum—a bistouri was made at one push to divide all the parts external to the director. This afforded ample space to admit of the forceps, and extraction of any calculus of moderate size, and it was already known, by the soundings, that there was none large. A director was now passed from the perineum into the bladder, and a scalpel passed in along the groove of the director, until I was satisfied I had made a division of sufficient extent. The forceps being introduced, a few calculi were removed; not finding any more, the fore-finger was introduced, and I soon discovered a very singular state of things. The prostate, or rather its middle lobe, together with the neck of the bladder, on its inside, were greatly thickened or enlarged, and projected a knob-like tumor into the bladder. The bas fond of the bladder had by some cause been pushed down between this projection and the rectum, so as to form a depression, or pocket, into which had fallen a great number of small calculi, and where, no doubt, these bodies had long been lodged, and where they were no doubt concealed during former soundings, and former operations, not those now present, but a succession of them, and I suppose it was only those that rose up out of the pocket, so as to come under the touch of the sound or the forceps that were felt or extracted. I proceeded to turn the point of my finger round the projection, so as to carry the point to the bottom, then straightening the finger, while I kept the upper side of the end of my finger, or nail, against each calculus, I could thus raise them out of the pocket, and pass them up into the upper part of the bladder, and there reach them with the forceps. I went on to raise a number at each introduction of the finger, and then removed them by the forceps. I proceeded until I was fully satisfied that I brought every stone out of the pocket. The forceps of Dr. Barton were used, and with them several of the calculi could be taken out at once. The operation finished, the patient was put to bed, much less exhausted than could have been expected. Indeed, he was greatly relieved by the operation, and until the termination of the third day, there were strong hopes of recovery. He was in a great degree freed from suffering, but the constitutional powers were worn down, and at the end of the fourth day Judge Sprigg's case terminated with his life.

Dr. Reynolds counted the calculi, and they were found to amount to forty-nine, besides a good many pieces—they might be resembled to a collection of Lima beans, supposing them to be thrown together from the smallest to the largest sizes of the ripe beans, and were equally flat.

The fact of there being now found such a number, so soon after two operations, goes to prove that the pocket above noticed had never been emptied before; besides the peculiar derangement of the bladder, renders it highly probable, that these calculi had, in greater part, been left undiscovered until the last operation.

It may confidently be asserted, that so far as the incisions were concerned, this was the least painful operation that was ever performed of its kind. One incision external, and one push of the scalpel into the bladder, constituted the whole of the cutting; and it is not a little curious that by one incision, the necessary and proper operations for fistula in ano, fistula in perineo, and the perinæum laid open as the first step of the operation of lithotomy, were all accomplished by one incision.

I would especially recommend, should it ever be the misfortune of a surgeon to cut the rectum any where near the sphincter ani, to divide that muscle as recommended by Mr. Copeland and Mr. Brodie, in cases of recto-vaginal fistulæ. And we feel assured that if the patient whose case has just been detailed, had had a little more strength to enable him to struggle against his disease, this simple method of treating his case would have cured the fistulæ; nor do I imagine that his life was shortened by the operation; on the contrary, there is much reason for believing, that he would have died two or three days sooner, had it not been performed, and would have suffered much more.

